# Crystal structure of 4,4′-(diazenediyl)dipyridinium nitrate perchlorate

**DOI:** 10.1107/S2056989022007885

**Published:** 2022-08-12

**Authors:** Qi-Ming Qiu, Jian-Biao Song, Ai-Guo Dong, Chuan-Tao Li, Zhi-Yuan Zheng

**Affiliations:** a School of Science, China University of Geosciences, Beijing 100083, People’s Republic of China; b Beijing Chaoyang Foreign Language School, Beijing 100101, People’s Republic of China; Moscow State University, Russia

**Keywords:** crystal structure, 4,4′-azo­pyridine, hydrogen bonds

## Abstract

The title compound, C_10_H_10_N_4_^2+^·NO_3_^−^·ClO_4_^−^, was obtained unexpectedly by the reaction of Co(ClO_4_)_2_·6H_2_O and cytidine-5′-monophosphate with 4,4′-azo­pyridine in an aqueous solution of nitric acid. The mol­ecular structure comprises two planar 4,4′-diazenediyldipyridinium dications lying on inversion centres and perchlorate and nitrate anions in general positions. In the crystal, N—H⋯O hydrogen bonds between dications and anions lead to the formation of [232] chains.

## Abstract 2

The title compound was obtained unexpectedly by the reaction of Co(ClO_4_)_2_·6H_2_O and cytidine-5′-monophosphate with 4,4′-azo­pyridine in an aqueous solution of nitric acid. In the crystal, N—H⋯O hydrogen bonds between dications and anions lead to the formation of chains.

## 1. Chemical context

If a mol­ecule contains two connected six-membered rings, and each of them contains N atoms, this mol­ecule can coordinate various metal ions in different ways. In particular, mol­ecules containing two or more pyridine rings are perfect bridging ligands to form supra­molecular structures (Zhang *et al.*, 2005 ▸; Rusu *et al.*, 2012 ▸; Theilmann *et al.*, 2009 ▸; Aakeröy *et al.*, 2013*a* ▸,*b* ▸; Huang *et al.*, 2016 ▸; Santana *et al.*, 2017 ▸; Hutchins *et al.*, 2018 ▸). In our previous work (Qiu *et al.*, 2017 ▸), we used, together with a cytidine-5′-monophosphate mononucleotide (CMP), an auxiliary ligands, namely 4,4′-azo­pyridine (azpy), to completely coordinate a metal ion to restrain the non-enzymatic hydrolysis of the phosphate group catalyzed by these ions, and we obtained the complex Co-CMP-azpy under pH = 5. As a result of the different charge states of CMP in aqueous solution, it seems to be meaningful to study nucleotide complexes at other pH values. Unexpectedly, single crystals of the title compound were obtained in a more acidic medium (pH = 3). The title compound is the first example of a salt of 4,4′-diazenediyldipyridinium dication with two different anions. 
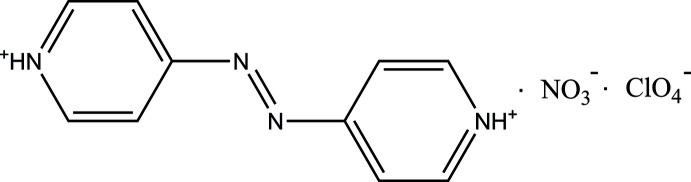


## 2. Structural commentary

**Figure 1:**
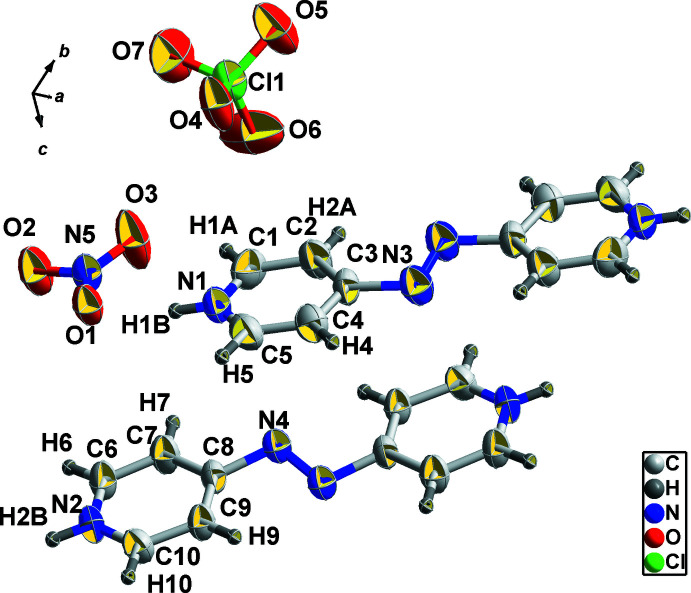
The structure of the title compound showing the asymmetric unit (labelled) with cations supplemented by the symmetry-generated moieties (not labelled) at 1 − *x*, 2 − *y*, 2 − *z* (molecule containing N1/N3) and at 1 − *x*, 1 − *y*, 2 − *z* (molecule containing N2/N4). Displacement ellipsoids are drawn at the 50% probability level.

The mol­ecular structure of the title compound comprises two planar [within 0.261 (5) Å] 4,4′-diazenediyldipyridinium dications lying on inversion centers and perchlorate and nitrate anions in general positions (Fig. 1 ▸). A planar conformation of the 4,4′-diazenediyldipyridinium dications is commonly observed for this type of compound. However, in the structure of bis­(4,4′-diazenediyldipyridinium) bis­(μ-chloro)­octa­chloro­dibismuth (POPHIO; Klein, 2019*a* ▸) pyridinium rings are twisted by 19.0 (4)°, whereas in the structure of 4,4′-diazenediyldipyridinium bis­(iodide) (POPKEN; Klein, 2019*b* ▸) the mean planes of the pyridinium rings form a dihedral angle of 84.1 (2)°. In the title 4,4’-diazenediyldipyridinium, the value of the dihedral angle between the planes passing through the pyridine rings is 0°.

## 3. Supra­molecular features

**Figure 2:**
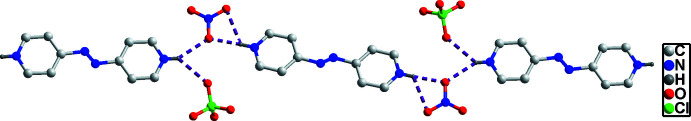
Chain in the structure of the title compound formed by N—H⋯O hydrogen bonds (shown as dashed lines). Hydrogen atoms not involved in hydrogen bonding are omitted.

The 4,4′-diazenediyldipyridinium dications are connected by N—H⋯O hydrogen bonds with nitrate anions thus forming chains directed along [232] (Fig. 2 ▸, Table 1 ▸). The perchlorate anions are attached to these chains *via* N—H⋯O hydrogen bonds. C—H⋯O inter­actions are also observed.

## 4. Database survey

A search of the Cambridge Structural Database (CSD version 5.40, update of March 2020; Groom *et al.*, 2016 ▸) for the 4,4′-diazenediyldipyridinium dication gave 17 hits, of which four purely organic structures are closely related to the title compound. In the crystal of 4,4′-diazenediyldipyridinium dinitrate (HUKQIN; Felloni *et al.*, 2002 ▸), N—H⋯O hydrogen bonds connect the dication to two anions, thus forming an island structure. The same type of structure is present in 4,4′-diazenediyldipyridinium dichloride (POPBUU; Klein, 2019*c* ▸) and 4,4′-diazenediyldipyridinium diiodide (POPKEN; Klein, 2019*b* ▸). In the salt with partially deprotonated 1,2,4,5-benzene­tetra­carb­oxy­lic acid (BULJEZ; Ravat *et al.*, 2015 ▸), the 4,4′-diazenediyldipyridinium dications act as the spacers that join the layers of hydrogen-bonded anions into a three-dimensional structure.

## 5. Synthesis and crystallization

An aqueous solution (5 mL) of cytidine-5′-monophosphate (32 mg, 0.10 mmol) was added to an aqueous solution (5 mL) of Co(ClO_4_)_2_·6H_2_O (18 mg, 0.05 mmol). After stirring for 10 min, 4,4′-azo­pyridine (9 mg, 0.05 mmol) in distilled water (5 mL) was added to this mixture. Nitric acid was also dropped to it and the resulting solution (pH = 3) was stirred at room temperature for 30 min. Red block-shaped crystals were obtained by evaporation at room temperature for two weeks.

## 6. Refinement

Crystal data, data collection and structure refinement details are summarized in Table 2 ▸. All H atoms were positioned geometrically (N—H = 0.86 Å, C—H = 0.93 Å) and refined using a riding model with *U*_iso_(H) = 1.2*U*_eq_(N,C).

## Supplementary Material

Crystal structure: contains datablock(s) I, global. DOI: 10.1107/S2056989022007885/yk2173sup1.cif

Structure factors: contains datablock(s) I. DOI: 10.1107/S2056989022007885/yk2173Isup2.hkl

CCDC reference: 2163316

Additional supporting information:  crystallographic information; 3D view; checkCIF report

## Figures and Tables

**Table 1:** Hydrogen-bond geometry (Å, °)

*D*—H⋯*A*	*D*—H	H⋯*A*	*D*⋯*A*	*D*—H⋯*A*
N2—H2*B*⋯O2^i^	0.86	2.47	3.046 (5)	125
N2—H2*B*⋯O1^i^	0.86	1.97	2.833 (4)	177
N2—H2*B*⋯N5^i^	0.86	2.58	3.372 (4)	153
N1—H1*B*⋯O4^ii^	0.86	2.42	3.078 (5)	134
N1—H1*B*⋯O1	0.86	2.22	2.989 (5)	150
C10—H10⋯O2^i^	0.93	2.46	3.049 (5)	122
C10—H10⋯O2^iii^	0.93	2.42	3.257 (5)	150
C9—H9⋯O7^iii^	0.93	2.58	3.191 (6)	124
C7—H7⋯O6^iv^	0.93	2.45	3.285 (6)	150
C6—H6⋯O5^v^	0.93	2.42	3.302 (6)	159
C6—H6⋯O4^v^	0.93	2.56	3.318 (5)	139
C6—H6⋯Cl1^v^	0.93	2.93	3.798 (4)	156
C5—H5⋯O7^ii^	0.93	2.53	3.447 (7)	169
C1—H1*A*⋯O3	0.93	2.28	3.117 (5)	150

Symmetry codes: (i) 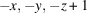 ; (ii) 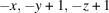 ; (iii) 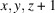 ; (iv) 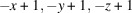 ; (v) 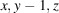 .

**Table 2:** Experimental details

Crystal data	
Chemical formula	C_10_H_10_N_4_^2+^·NO_3_^−^·ClO_4_^−^
*M*_r_	347.68
Crystal system, space group	Triclinic, *P* 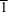
Temperature (K)	298
*a*, *b*, *c* (Å)	8.3023 (8), 10.0792 (9), 10.1052 (9)
α, β, γ (°)	116.966 (3), 105.481 (2), 92.871 (1)
*V* (Å^3^)	711.77 (11)
*Z*	2
Radiation type	Mo *K*α
μ (mm^−1^)	0.32
Crystal size (mm)	0.45 × 0.40 × 0.33

Data collection	
Diffractometer	Bruker APEXII CCD
Absorption correction	Multi-scan (*SADABS*; Krause *et al.*, 2015 ▸)
*T*_min_, *T*_max_	0.873, 0.904
No. of measured, independent and observed [*I* > 2σ(*I*)] reflections	3625, 2466, 1896
*R*_int_	0.034
(sin θ/λ)_max_ (Å^−1^)	0.596

Refinement	
*R*[*F* ^2^ > 2σ(*F* ^2^)], *wR*(*F* ^2^), *S*	0.081, 0.250, 1.00
No. of reflections	2466
No. of parameters	208
H-atom treatment	H-atom parameters constrained
Δρ_max_, Δρ_min_ (e Å^−3^)	0.63, −0.51

Computer programs: *APEX2* and *SAINT* (Bruker, 2006 ▸), *SHELXT* (Sheldrick, 2015*a* ▸), *SHELXL2018/3* (Sheldrick, 2015*b* ▸), *SHELXTL* (Sheldrick, 2008 ▸).

## supplementary crystallographic information

### Crystal data

**Table:**

C_10_H_10_N_4_^2+^·NO_3_^−^·ClO_4_^−^	*Z* = 2
*M**_r_* = 347.68	*F*(000) = 356
Triclinic, *P*1	*D*_x_ = 1.622 Mg m^−^^3^
*a* = 8.3023 (8) Å	Mo *K*α radiation, λ = 0.71073 Å
*b* = 10.0792 (9) Å	Cell parameters from 1709 reflections
*c* = 10.1052 (9) Å	θ = 2.3–25.3°
α = 116.966 (3)°	µ = 0.32 mm^−^^1^
β = 105.481 (2)°	*T* = 298 K
γ = 92.871 (1)°	Block, red
*V* = 711.77 (11) Å^3^	0.45 × 0.40 × 0.33 mm

### Data collection

**Table:**

Bruker APEXII CCD diffractometer	1896 reflections with *I* > 2σ(*I*)
Radiation source: fine-focus sealed tube, Bruker (Mo) X-ray Source	*R*_int_ = 0.034
phi and ω continuous scans	θ_max_ = 25.1°, θ_min_ = 2.3°
Absorption correction: multi-scan (SADABS; Krause *et al.*, 2015)	*h* = −9→9
*T*_min_ = 0.873, *T*_max_ = 0.904	*k* = −11→7
3625 measured reflections	*l* = −12→12
2466 independent reflections	

### Refinement

**Table:**

Refinement on *F*^2^	0 restraints
Least-squares matrix: full	Hydrogen site location: inferred from neighbouring sites
*R*[*F*^2^ > 2σ(*F*^2^)] = 0.081	H-atom parameters constrained
*wR*(*F*^2^) = 0.250	*w* = 1/[σ^2^(*F*_o_^2^) + (0.189*P*)^2^ + 0.2213*P*] where *P* = (*F*_o_^2^ + 2*F*_c_^2^)/3
*S* = 1.00	(Δ/σ)_max_ < 0.001
2466 reflections	Δρ_max_ = 0.63 e Å^−^^3^
208 parameters	Δρ_min_ = −0.51 e Å^−^^3^

### Special details

**Table:**

Geometry. All esds (except the esd in the dihedral angle between two l.s. planes) are estimated using the full covariance matrix. The cell esds are taken into account individually in the estimation of esds in distances, angles and torsion angles; correlations between esds in cell parameters are only used when they are defined by crystal symmetry. An approximate (isotropic) treatment of cell esds is used for estimating esds involving l.s. planes.

### Fractional atomic coordinates and isotropic or equivalent isotropic displacement parameters (Å^2^)

**Table:**

	*x*	*y*	*z*	*U*_iso_*/*U*_eq_	
C1	0.2913 (7)	0.5983 (5)	0.6432 (5)	0.0737 (14)	
H1A	0.305031	0.544442	0.545363	0.088*	
C2	0.3896 (6)	0.7426 (5)	0.7501 (5)	0.0611 (11)	
H2A	0.469672	0.786844	0.725164	0.073*	
C3	0.3655 (5)	0.8180 (4)	0.8929 (4)	0.0494 (9)	
C4	0.2457 (5)	0.7514 (5)	0.9279 (5)	0.0583 (10)	
H4	0.228689	0.802795	1.024639	0.070*	
C5	0.1525 (6)	0.6100 (6)	0.8199 (6)	0.0681 (13)	
H5	0.071484	0.563924	0.842278	0.082*	
C6	0.2190 (5)	0.0818 (4)	0.6583 (4)	0.0539 (10)	
H6	0.201694	0.007099	0.555676	0.065*	
C7	0.3250 (5)	0.2196 (5)	0.7198 (4)	0.0510 (9)	
H7	0.377227	0.240802	0.658865	0.061*	
C8	0.3518 (4)	0.3251 (4)	0.8734 (4)	0.0424 (8)	
C9	0.2710 (5)	0.2956 (4)	0.9628 (4)	0.0465 (9)	
H9	0.288421	0.367714	1.066539	0.056*	
C10	0.1631 (5)	0.1558 (4)	0.8943 (5)	0.0513 (9)	
H10	0.106801	0.132813	0.951752	0.062*	
N1	0.1780 (6)	0.5378 (4)	0.6816 (5)	0.0723 (12)	
H1B	0.118526	0.448315	0.614476	0.087*	
N2	0.1408 (4)	0.0552 (3)	0.7461 (4)	0.0510 (8)	
H2B	0.073137	−0.030783	0.704763	0.061*	
N3	0.4662 (5)	0.9644 (4)	1.0220 (4)	0.0617 (9)	
N4	0.4601 (4)	0.4702 (3)	0.9303 (3)	0.0464 (8)	
N5	0.1514 (4)	0.2180 (4)	0.2959 (4)	0.0529 (8)	
O1	0.0745 (4)	0.2327 (3)	0.3941 (3)	0.0592 (8)	
O2	0.1175 (5)	0.0958 (4)	0.1741 (4)	0.0848 (11)	
O3	0.2412 (6)	0.3270 (4)	0.3114 (4)	0.1011 (15)	
Cl1	0.22108 (13)	0.71463 (11)	0.30697 (11)	0.0595 (4)	
O4	0.0952 (4)	0.7204 (4)	0.3809 (4)	0.0798 (10)	
O5	0.2642 (6)	0.8577 (4)	0.3160 (5)	0.1046 (14)	
O6	0.3646 (6)	0.6719 (7)	0.3762 (7)	0.131 (2)	
O7	0.1536 (7)	0.6059 (5)	0.1466 (5)	0.1112 (15)	

### Atomic displacement parameters (Å^2^)

**Table:**

	*U*^11^	*U*^22^	*U*^33^	*U*^12^	*U*^13^	*U*^23^
C1	0.106 (4)	0.049 (3)	0.042 (2)	0.015 (3)	0.015 (2)	0.007 (2)
C2	0.072 (3)	0.045 (2)	0.057 (2)	0.004 (2)	0.026 (2)	0.0163 (19)
C3	0.047 (2)	0.0333 (18)	0.051 (2)	0.0021 (15)	0.0095 (16)	0.0102 (16)
C4	0.052 (2)	0.054 (2)	0.064 (2)	0.0061 (18)	0.0195 (18)	0.025 (2)
C5	0.054 (3)	0.056 (3)	0.091 (4)	−0.001 (2)	0.009 (2)	0.043 (3)
C6	0.058 (2)	0.041 (2)	0.0427 (19)	−0.0034 (17)	0.0141 (17)	0.0061 (16)
C7	0.048 (2)	0.048 (2)	0.050 (2)	−0.0002 (16)	0.0212 (16)	0.0161 (18)
C8	0.0385 (18)	0.0290 (16)	0.0469 (19)	0.0011 (13)	0.0086 (14)	0.0114 (15)
C9	0.051 (2)	0.0363 (18)	0.0387 (18)	0.0029 (15)	0.0151 (15)	0.0075 (15)
C10	0.055 (2)	0.044 (2)	0.057 (2)	0.0035 (17)	0.0230 (17)	0.0241 (18)
N1	0.076 (3)	0.0365 (18)	0.072 (3)	−0.0058 (17)	−0.013 (2)	0.0219 (19)
N2	0.0477 (18)	0.0341 (16)	0.0548 (18)	−0.0064 (13)	0.0102 (14)	0.0134 (15)
N3	0.074 (3)	0.050 (2)	0.057 (2)	0.0032 (17)	0.0302 (17)	0.0182 (17)
N4	0.0490 (18)	0.0371 (16)	0.0453 (15)	0.0039 (13)	0.0147 (13)	0.0144 (14)
N5	0.058 (2)	0.0440 (18)	0.0464 (17)	−0.0044 (15)	0.0227 (14)	0.0116 (15)
O1	0.0702 (19)	0.0487 (16)	0.0513 (15)	−0.0080 (13)	0.0297 (14)	0.0148 (13)
O2	0.122 (3)	0.0550 (19)	0.0581 (18)	−0.0123 (18)	0.0462 (19)	0.0060 (15)
O3	0.121 (3)	0.067 (2)	0.092 (3)	−0.031 (2)	0.065 (2)	0.0067 (19)
Cl1	0.0639 (7)	0.0527 (7)	0.0564 (7)	0.0023 (5)	0.0317 (5)	0.0162 (5)
O4	0.078 (2)	0.0570 (19)	0.092 (2)	−0.0099 (15)	0.0561 (19)	0.0123 (17)
O5	0.147 (4)	0.063 (2)	0.116 (3)	0.003 (2)	0.078 (3)	0.036 (2)
O6	0.087 (3)	0.209 (6)	0.172 (5)	0.040 (3)	0.057 (3)	0.145 (5)
O7	0.162 (4)	0.081 (3)	0.070 (2)	0.012 (3)	0.059 (3)	0.010 (2)

### Geometric parameters (Å, º)

**Table:**

C1—N1	1.326 (7)	C8—N4	1.452 (4)
C1—C2	1.391 (6)	C9—C10	1.388 (5)
C1—H1A	0.9300	C9—H9	0.9300
C2—C3	1.369 (6)	C10—N2	1.327 (5)
C2—H2A	0.9300	C10—H10	0.9300
C3—C4	1.379 (6)	N1—H1B	0.8600
C3—N3	1.458 (5)	N2—H2B	0.8600
C4—C5	1.360 (6)	N3—N3^i^	1.188 (7)
C4—H4	0.9300	N4—N4^ii^	1.216 (6)
C5—N1	1.334 (7)	N5—O3	1.219 (4)
C5—H5	0.9300	N5—O2	1.232 (4)
C6—N2	1.335 (5)	N5—O1	1.276 (4)
C6—C7	1.377 (6)	Cl1—O6	1.405 (5)
C6—H6	0.9300	Cl1—O7	1.408 (4)
C7—C8	1.373 (5)	Cl1—O5	1.424 (4)
C7—H7	0.9300	Cl1—O4	1.427 (3)
C8—C9	1.379 (5)		
			
N1—C1—C2	119.7 (4)	C8—C9—C10	118.4 (3)
N1—C1—H1A	120.2	C8—C9—H9	120.8
C2—C1—H1A	120.2	C10—C9—H9	120.8
C3—C2—C1	118.3 (4)	N2—C10—C9	119.4 (3)
C3—C2—H2A	120.9	N2—C10—H10	120.3
C1—C2—H2A	120.9	C9—C10—H10	120.3
C2—C3—C4	120.2 (4)	C1—N1—C5	122.8 (4)
C2—C3—N3	125.2 (4)	C1—N1—H1B	118.6
C4—C3—N3	114.4 (3)	C5—N1—H1B	118.6
C5—C4—C3	119.4 (4)	C10—N2—C6	122.9 (3)
C5—C4—H4	120.3	C10—N2—H2B	118.6
C3—C4—H4	120.3	C6—N2—H2B	118.6
N1—C5—C4	119.6 (5)	N3^i^—N3—C3	112.2 (4)
N1—C5—H5	120.2	N4^ii^—N4—C8	112.6 (4)
C4—C5—H5	120.2	O3—N5—O2	119.4 (3)
N2—C6—C7	120.0 (3)	O3—N5—O1	121.1 (3)
N2—C6—H6	120.0	O2—N5—O1	118.9 (3)
C7—C6—H6	120.0	O6—Cl1—O7	108.4 (3)
C8—C7—C6	118.3 (4)	O6—Cl1—O5	111.8 (3)
C8—C7—H7	120.9	O7—Cl1—O5	107.3 (3)
C6—C7—H7	120.9	O6—Cl1—O4	110.1 (2)
C7—C8—C9	121.0 (3)	O7—Cl1—O4	109.5 (3)
C7—C8—N4	115.9 (3)	O5—Cl1—O4	109.7 (2)
C9—C8—N4	123.0 (3)		
			
N1—C1—C2—C3	0.0 (7)	N4—C8—C9—C10	176.6 (3)
C1—C2—C3—C4	−0.4 (6)	C8—C9—C10—N2	0.0 (6)
C1—C2—C3—N3	175.1 (4)	C2—C1—N1—C5	0.3 (7)
C2—C3—C4—C5	0.4 (6)	C4—C5—N1—C1	−0.2 (7)
N3—C3—C4—C5	−175.6 (4)	C9—C10—N2—C6	0.4 (6)
C3—C4—C5—N1	−0.1 (6)	C7—C6—N2—C10	−1.5 (6)
N2—C6—C7—C8	2.2 (6)	C2—C3—N3—N3^i^	22.8 (7)
C6—C7—C8—C9	−1.8 (6)	C4—C3—N3—N3^i^	−161.4 (5)
C6—C7—C8—N4	−178.0 (3)	C7—C8—N4—N4^ii^	−149.2 (4)
C7—C8—C9—C10	0.7 (5)	C9—C8—N4—N4^ii^	34.8 (5)

Symmetry codes: (i) −*x*+1, −*y*+2, −*z*+2; (ii) −*x*+1, −*y*+1, −*z*+2.

### Hydrogen-bond geometry (Å, º)

**Table:**

*D*—H···*A*	*D*—H	H···*A*	*D*···*A*	*D*—H···*A*
N2—H2*B*···O2^iii^	0.86	2.47	3.046 (5)	125
N2—H2*B*···O1^iii^	0.86	1.97	2.833 (4)	177
N2—H2*B*···N5^iii^	0.86	2.58	3.372 (4)	153
N1—H1*B*···O4^iv^	0.86	2.42	3.078 (5)	134
N1—H1*B*···O1	0.86	2.22	2.989 (5)	150
C10—H10···O2^iii^	0.93	2.46	3.049 (5)	122
C10—H10···O2^v^	0.93	2.42	3.257 (5)	150
C9—H9···O7^v^	0.93	2.58	3.191 (6)	124
C7—H7···O6^vi^	0.93	2.45	3.285 (6)	150
C6—H6···O5^vii^	0.93	2.42	3.302 (6)	159
C6—H6···O4^vii^	0.93	2.56	3.318 (5)	139
C6—H6···Cl1^vii^	0.93	2.93	3.798 (4)	156
C5—H5···O7^iv^	0.93	2.53	3.447 (7)	169
C1—H1*A*···O3	0.93	2.28	3.117 (5)	150

Symmetry codes: (iii) −*x*, −*y*, −*z*+1; (iv) −*x*, −*y*+1, −*z*+1; (v) *x*, *y*, *z*+1; (vi) −*x*+1, −*y*+1, −*z*+1; (vii) *x*, *y*−1, *z*.
